# Incidence and risk factors of pancreatic cancer during 15 years follow-up in the Golestan Cohort Study in Iran

**DOI:** 10.1371/journal.pone.0300736

**Published:** 2024-06-07

**Authors:** Sara Mirzamohamadi, Mohammad Navid HajiAbbasi, Gholamreza Roshandel, Mehdi Alimadadi, Seyed Behzad Mirheidari, Somayeh Ghorbani, Akram Pourshams, Maryam Zahedi

**Affiliations:** 1 Tehran University of Medical Sciences, Tehran, Iran; 2 Isfahan University of Medical Sciences, Isfahan, Iran; 3 Golestan Research Center of Gastroenterology and Hepatology, Golestan University of Medical Sciences, Gorgan, Iran; 4 Golestan University of Medical Sciences, Gorgan, Iran; 5 Cancer Research Center, Golestan University of Medical Sciences, Gorgan, Iran; 6 Digestive Oncology Research Center, Digestive Diseases Research Institute, Tehran University of Medical Sciences, Tehran, Iran; 7 Department of Internal medicine, Endocrinology and Metabolic disorders, Clinical Research Development Unit (CRDU), Sayad Shirazi Hospital, Golestan University of Medical Sciences, Gorgan, Iran; Howard University, UNITED STATES

## Abstract

**Background:**

Cancer is one of the main causes of death in the worldwide. Pancreatic Cancer (PC) is prevalent in developed and increasing in developing countries. PC is important because of its low survival rate, high fatality, and increasing incidence. Therefore, identifying risk factors to prevent its development is necessary. This study aimed to determine incidence of PC and its risk factors in the Golestan Cohort Study (GCS) in Iran.

**Method:**

This study is a prospective population-based cohort study in the frame of GCS with 15 years of follow-up for PC. GCS was launched in the Golestan province of Iran with 50045 participants who were 40 to 75 years old. variables included: age, gender, education status, smoking, alcohol consumption, opium usage, type of blood group, dyslipidemia, body mass index (BMI), waist circumference (WC), family history (FH) of PC, ethnicity, and history of diabetes mellitus (DM).

**Result:**

Among 50045 participants of GCS during 15 years of follow up, 100 people were diagnosed PC. PC incidence was 0.2%. Age-standardized incidence rate (ASR) of PC in the study population was 11.12 per 100,000 person-years. People with age ≥60 years were 46, in 50–59 years old group were 36, and 18 of them were <50 years (p<0.001). The smoking rate in PC group was 27% (p<0.01). Univariate model of cox regression analysis showed age 50–59, ≥60 years compared to <50 years [HR:3.006, 95%CI (1.707–5.294), p<0.001], [HR: 6.727, 95% CI (3.899–11.608), p<0.001], male gender [HR:1.541, 95%CI (1.041–2.281), p = 0.031], opium use [HR:1.436, 95% CI (0.887–2.324), p = 0.141], and smoking [HR:1.884, 95%CI (1.211–2.929), p = 0.005] were predictors for PC. In the multivariate model after adjusting, age 50–59 [HR:2.99, 95% CI (1.698–5.265), p<0.001], and ≥60 years [HR: 6.564, 95% CI (3.797–11.346), p<0.001] was the only predictor for PC.

**Conclusion:**

This study revealed an incidence of PC 0.2% in GCS in Iran. Main risk factor for PC was older age.

## Introduction

Cancer is a leading cause of mortality worldwide. Based on 2020 reports, approximately 19 million new cases and 10 million deaths in terms of the cancer were recorded [1]. The seventh disease with the highest fatality rate, pancreatic cancer (PC), is more common in industrialized nations [2]. Around 466,000 PC-related deaths are anticipated globally in 2020. This disease is a significant public health problem, and one of the ten most common causes of death from cancer in 130 countries worldwide [3]. The incidence of PC globally increased 2.3 times in both sexes from 1990 to 2017 [4]. In almost all countries, PC prevalence and mortality are increasing [4]. The highest incidence and mortality from PC are in high-income countries, although PC causes are still unknown [4]. PC is still one of deadliest malignancies; despite the advancements in the diagnosis, and cure of PC in last decade, the average five-year survival rate is still only about 9 percent [2].

According to GLOBOCAN 2020 report, PC was the 14ththe most common cancer in Iran, with an incidence of 2.4% and a mortality rate of 3.9% [5]. Sheikh et al. showed a median survival 6.2 month, a one year survival rate 26.2%, and a five year 1.5% in the patients with PC in Iran [6].

PC development is influenced by both environmental and genetic factors, much like other malignancies [7]. Because various age groups have varied biological and socioeconomic consequences, PC risk factors are complicated and multidimensional [8]. Various risk factors have been described in further epidemiological studies, and their impact on PC is still debated [7]. In some studies, smoking, aging, diabetes mellitus (DM), drinking alcohol, obesity, family history of PC and chronic pancreatitis are among main risk factors [7, 9].

The highest prevalence of PC related to the age of 50 years and after [8], and in terms of the growth and increase in population, and life expectancy, a dramatic increase in the prevalence of PC in low-income and middle-income countries is expected in the future [4].

PC has few and diverse symptoms, which delays diagnosis. As a result, the most PC patients were referred when they were very sick [9]. PC is almost hard to detect early. Depending on where the tumor is, some of these symptoms may exist. The tumor’s location is mainly limited to the head of pancreas, which can spread to other places and cause jaundice, which results in yellowing of skin and eyes, as well as weight loss. Some significant early symptoms include weight loss, abdominal pain, and satiety [10].

Considering the gap in the available information, making appropriate decisions for this outbreak is a big challenge, and the need to identify risk factors related to the age of onset of PC can help allocate resources effectively for prevention.

The Golestan Cohort Study (GCS) was carried out in the Golestan region of Iran 15 years ago to identify environmental and genetic variables related to esophageal cancer. This study aimed to determine the incidence of PC, and its associated risk factors in the GCS in Iran and identify whether PC as a cancer related to the digestive system cancers had a high incidence in this area or not.

## Method

### Study design

This study was a prospective population-based cohort study that conducted in the GCS. The GCS was launched in the Golestan province of Iran. Participants including 50,045 people 40 to 75 years old were recruited between 2004 and 2008. Participants included 39399 rural people from 326 villages and 10645 urban people. All of them followed up for 15 years for PC incidence.

### Data collection

In the present study, variables included: age, gender, education status, alcohol consumption, opium use, smoking, body mass index (BMI), blood groups, dyslipidemia, waist circumference (WC), family history (FH) of PC, ethnicity, and history of DM.

Demographics characteristics included age, sex, literacy, ethnicity, FH, past medical history (PMH), drug history (DH), and smoke, alcohol, and opium usage collected through the interview with participants. WC, weight, and height measured by a trained technician.

Age is categorized as <50 and ≥50 years. Education is classified as illiterate and literate. The blood group included A, B, AB, O. Dyslipidemia was considered as every participant used anti-lipid medication. At the baseline of the GCS, the diagnosis of DM was based on the history of anti-diabetes drugs (ATD) use or self-reported previous physician diagnosis. BMI is calculated by dividing measured weight (kg) by the square of the estimated height (m^2^). It was then categorized into two groups: underweight and normal (BMI<18.5 kg/m^2^ and 18.5<BMI<25 kg/m^2^) versus overweight and obese (25<BMI<30kg/m^2^ and BMI>30 kg/m^2^). WC is organized into two groups: standard or high risk. According to the adult treatment panel (ATP) III criteria people with WC>102 cm in men and >88 cm in women were high-risk [11]. Ethnicity is categorized as Turkman and others (Fars, Turk, Sistani, Baloch, Kurd, Afghani). People who smoked cigarettes or other substances (nass, hookah, and pipe) with one or more times a week for at least six months or more are considered smoker. Participants were divided into two groups: no smokers and current or former smokers. Opium users were individuals who used opium or heroin at least once a week for six months or more. Alcohol consumption is considered as consummated beer, homemade wine, or imported wines at least once a month for six months or more. Participants followed annually to capture the study outcome (PC).

### Statistical analyses

The nominal or categorical variables are compared by cross-tabulation between PC and non-PC groups. The relationship between risk factors and PC was evaluated by univariable and multivariable cox regression analysis. Also, time-to-event curves were generated by Kaplan-Meier analysis. Crude and adjusted hazard ratio (HR) and 95% confidence interval (CI) were calculated. For time-to-event calculation, we considered age as the timescale and the entry time defined as the participant’s age at enrolment in the GCS. The time to an event considered from the time of entering the study to the incidence of PC. Others followed up until the end of the study or until death in terms of outcome (PC). Statistical significance was accepted at p-values < 0.05 for the cross-tabulation test and multivariate cox regression. However, in the univariate model of cox regression all variables with p-value <0.2 were considered statistically significant and are entered in the multivariate model. Data analysis done using SPSS 26.

### Ethics approval

The study protocol and the informed consent used for Golestan Cohort Study (GCS) were approved by the ethical review committees of the Digestive Disease Research Center, Shariati Hospital, Tehran University of Medical Sciences, Tehran, Iran (DDRC), International Agency for Research on Cancer, Lyon, France (IARC) and Division of Cancer Epidemiology and Genetics, National Cancer Institute, Bethesda, MD, USA (NCI).

## Result

**Fig 1:**
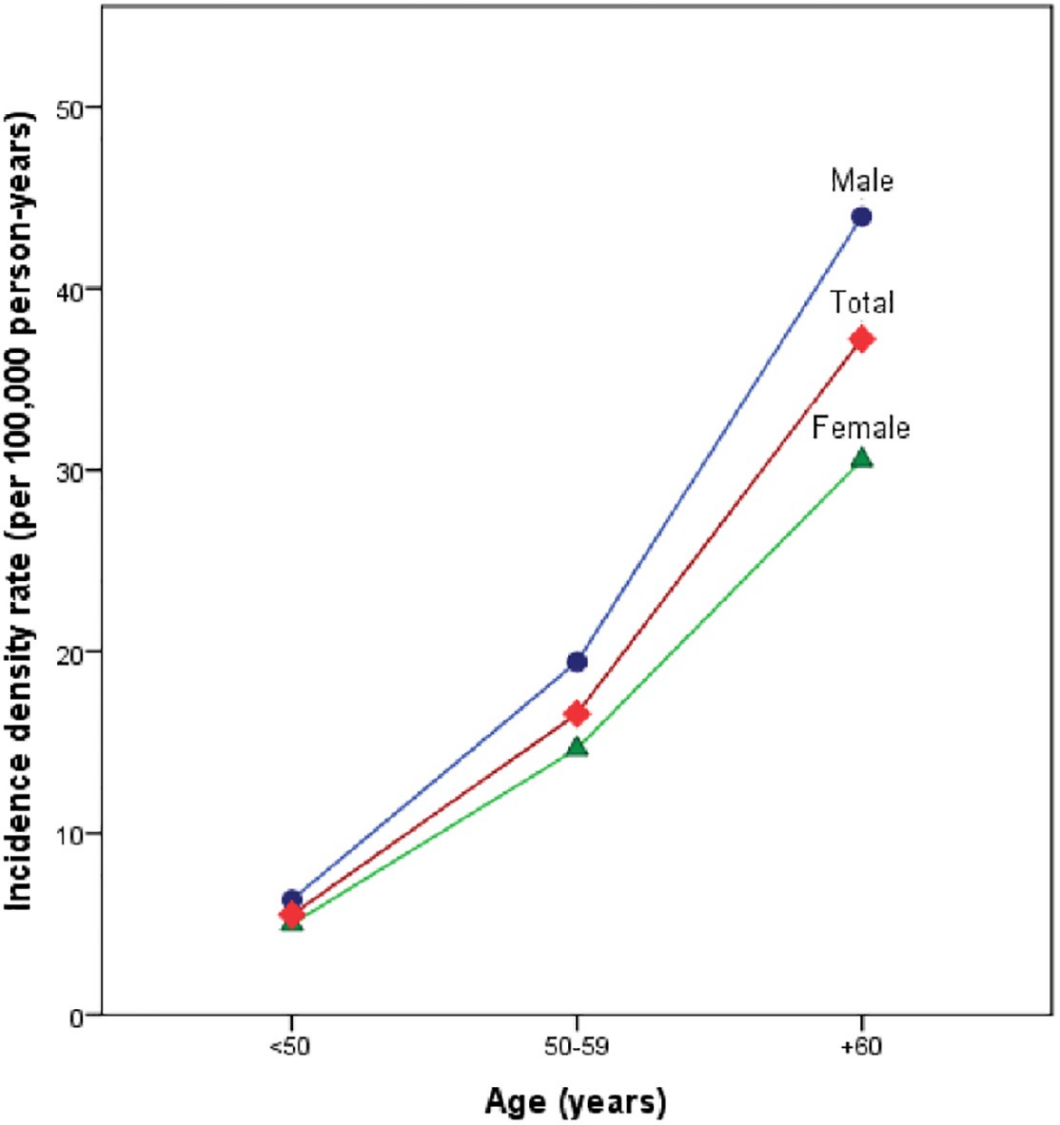
Incidence density by age and gender.

Among 50045 participants of GCS during 15 years of follow-up, 100 people reported PC. PC incidence was 0.2%. Cumulative incidence per 10,000 people were 7.79 in <50 years, 22.37 in 50–59 years old group, and 42.8 in ≥60 years. Also, incidence density per 100,000 person-year were higher in men rather than women (18.82 vs. 12.23) and in ≥60 years age group rather than other age groups (37.22 in ≥60 vs. 16.57 in 50–59 and 5.51 in<50 years). Age-standardized incidence rate (ASR) of PC in the study population was 11.12 per 100,000 person-years Tables 1 and 2, and Fig 1.

**Table 1:** Cumulative incidence by gender and age group at the time of study enrollment.

Sex	Age at enrollment	No. of Pancreatic Cancer Cases	No. of Participants	Cumulative incidence per 10,000
Male	< 50.0	8	9018	8.871
	50.0–59.0	17	6614	25.70
	60.0+	27	5549	48.66
Female	< 50.0	10	14082	7.10
	50.0–59.0	19	9482	20.04
	60.0+	19	5199	36.55
Total	< 50.0	18	23100	7.79
	50.0–59.0	36	16096	22.37
	60.0+	46	10748	42.80

**Table 2:** Incidence density in general and by gender and age group at the time of study enrollment.

Variable	No. of Pancreatic Cancer Cases	Person-year	Incidence density per 100,000 person-years
Gender			
Male	52	275324	18.89
Female	48	392483	12.23
Age			
<50	18	326922	5.51
50–59	36	217282	16.57
+60	46	123603	37.22
Gender			
Male			
<50	8	126361	6.33
50–59	17	87549	19.42
+60	27	61414	43.96
Female			
<50	10	200561	4.99
50–59	19	129733	14.65
+60	19	62189	30.55
Total	100	667807	14.97

The baseline and clinical characteristics of participants compared between PC and non-PC groups are presented in Table 3. PC group included 52 (52%) men, and 48 (48%) women. People with age ≥60 years were 46, in 50–59 years old group were 36, and 18 of them were <50 years (p<0.001). Seventy-three people in the PC group were illiterate, and 27 of them were literate. Forty-one people had underweight and normal BMI, and 59 others were overweight and obese in the PC group. Only one person in the PC group had dyslipidemia. Five people had a DM history in this group. Twenty-one people were current opium users, and five of them were alcohol users. Twenty-seven participants were current smokers (p = 0.01). None of the people in the PC group had an FH of PC, whereas, in the non-PC group, 37 people reported an FH of PC, that none of them developed PC in follow-up years. Seventy-five people in the PC group were Turkman; also 74.29% of all participants in GCS were Turkman. Blood groups in the PC group included A in 34 participants, B in 32, AB in 6 people, and O in 28 of them, respectively.

**Table 3:** Baseline characteristics of pancreatic cancer patients and non-pancreatic cancer, N (%).

Variables	PC group	Non-PC group	P-value
	N = 100	N = 49944	
**Gender**			0.053
** Male**	52 (52)	21181 (42)	
** Female**	48 (48)	28763 (57)	
**Age (year)**			**<0.001**
** <50**	18 (18)	23100 (46.3)	
** 50–59**	36 (36)	16096 (32.2)	
** ≥60**	46 (46)	10748 (21.5)	
**Education**			0.536
** Illiterate**	73 (73)	35045 (70)	
** Literate**	27 (27)	14899 (29)	
**Ethnicity**			0.898
** Turkman**	75 (75)	37178 (74)	
** Others**	25 (25)	12766 (25)	
**WC**			0.79
**Standard**	48 (48)	23309 (46)	
**High risk**	52 (52)	26634 (53)	
**BMI**			0.93
** Underweight-normal**	41 (41)	20257 (40)	
** Overweight-obese**	59 (59)	29679 (59)	
**DLP**	1 (1)	838 (1.6)	>0.999
**Opium use**	21 (21)	8464 (16)	0.281
**DM**	5 (5)	3439 (6.8)	0.457
**Smoking**	27 (27)	8629 (17)	**0.01**
**Alcohol consumption**	5 (5)	1723 (3.4)	0.4
**FH of PC**	0 (0)	37 (0.07)	>0.999
**BG**			0.527
** A**	34 (34)	16671 (33)	
** B**	32 (32)	13523 (27)	
** AB**	6 (6)	4804 (9.6)	
** O**	28 (28)	14942 (29)	

PC: pancreatic cancer; WC: waist circumference; BMI: body mass index; DLP: dyslipidemia; DM: diabetes mellitus; FH: family history of pancreatic cancer; BG: blood group

Univariate model of cox regression analysis showed age 50–59, ≥60 years compared to <50 years [HR:3.006, 95%CI (1.707–5.294), p<0.001], [HR: 6.727, 95% CI (3.899–11.608), p<0.001], male gender [HR:1.541, 95%CI (1.041–2.281), p = 0.031], opium use [HR:1.436, 95% CI (0.887–2.324), p = 0.141], and smoking [HR:1.884, 95%CI (1.211–2.929), p = 0.005] were predictors for PC. The multivariate model demonstrated that age 50–59 years increased PC chance 2.99 times compared to the age < 50 years [HR:2.99, 95% CI (1.698–5.265), p<0.001] and age ≥60 years increased PC developing 6.564 times compared to age <50 years [HR: 6.564, 95% CI (3.797–11.346), p<0.001]. Then, age ≥ 50 years was the only predictor for PC after adjusting for gender, opium, and smoking (Table 4).

**Table 4:** Crude and adjusted hazard ratio (HR) and 95% confidence intervals (CI) of pancreatic cancer risk factors.

Variables	Univariate			Multivariate		
	HR	95%CI	P-value	HR	95% CI	P-value
**Age**						
**<50**	References		<0.001	Reference		
**50–59**	3.006	1.707–5.294		2.99	1.698–5.265	**<0.001**
**≥60**	6.727	3.899–11.608		6.564	3.797–11.346	
**Gender**						
**female**	References			References		
**male**	1.541	1.041–2.281	0.031	1.099	0.691–1.747	0.69
**opium**						
**never user**	References			References		
**ever user**	1.436	0.887–2.324	0.141	1.004	0.589–1.713	0.987
**Smoking**						
**never user**	References			References		
**ever user**	1.884	1.211–2.929	0.005	1.694	0.976–2.942	0.061
**Education**						
**Literate**	References					
**Illiterate**	1.194	0.768–1.856	0.432			
**BMI**						
**Underweight-normal**	References					
**overweight/obese**	1.057	0.709–1.574	0.786			
**DM**	0.795	0.324–1.955	0.618			
**Alcohol**						
**never user**	References					
**ever user**	1.536	0.625–3.776	0.349			
**FH**	20.109	0.0–8.661	0.851			
**Ethnicity**						
**non-Turkman**	References					
**Turkman**	1.002	0.637–1.575	0.994			
**BG**						
**A**	Reference					
**B**	1.162	0.717–1.882	0.543			
**AB**	0.612	0.257–1.457	0.267			
**O**	0.915	0.555–1.509	0.728			
**DLP**	1.624	0.226–11.642	0.63			
**WC**						
**high-risk**	References					
**Standard**	0.921	0.622–1.364	0.681			

BMI: body mass index; DM: diabetes mellitus; FH: family history; BG: blood group; DLP: dyslipidemia; WC: waist circumference

**Fig 2:**
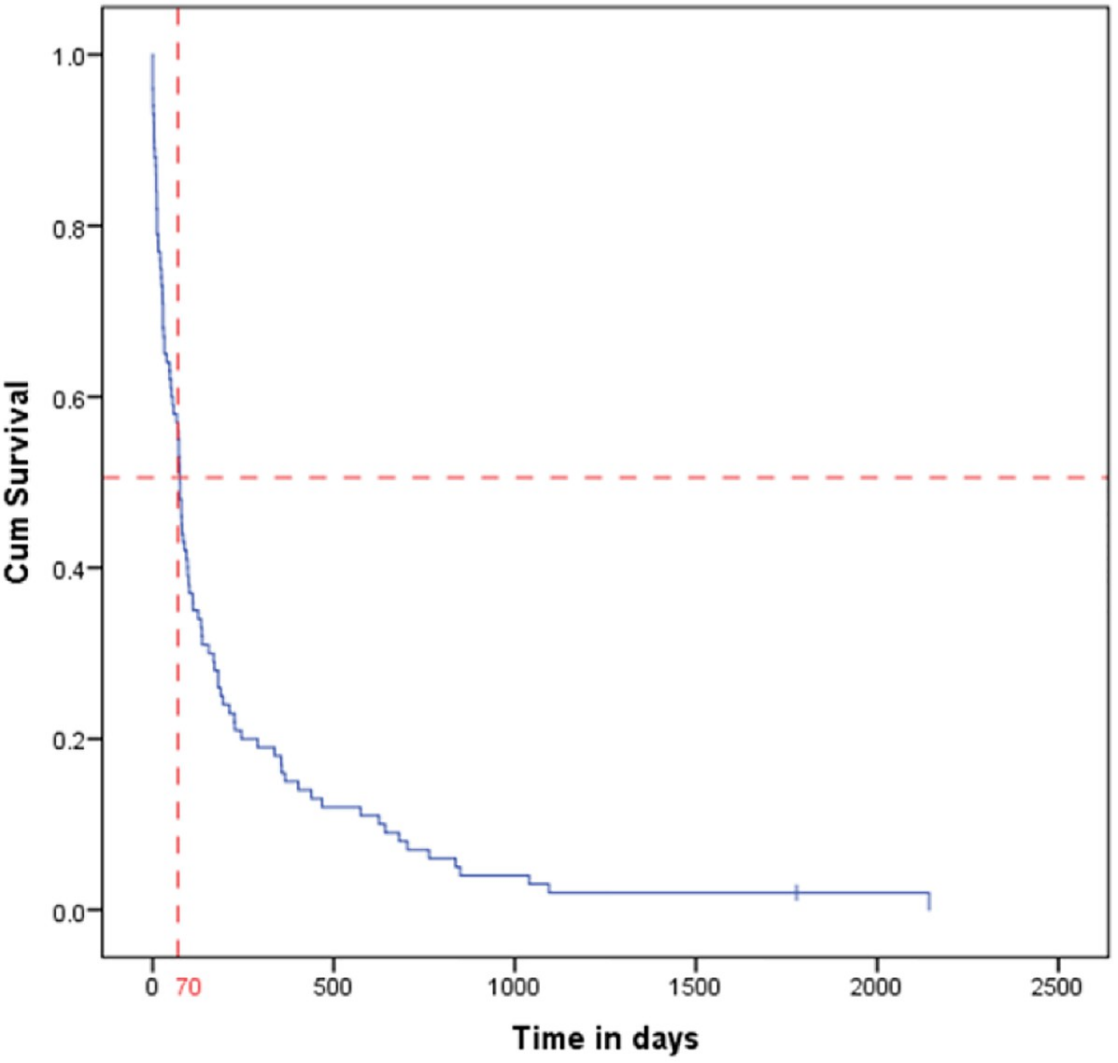
Kaplan-Meier curve for pancreatic cancer participants survival.

**Fig 3:**
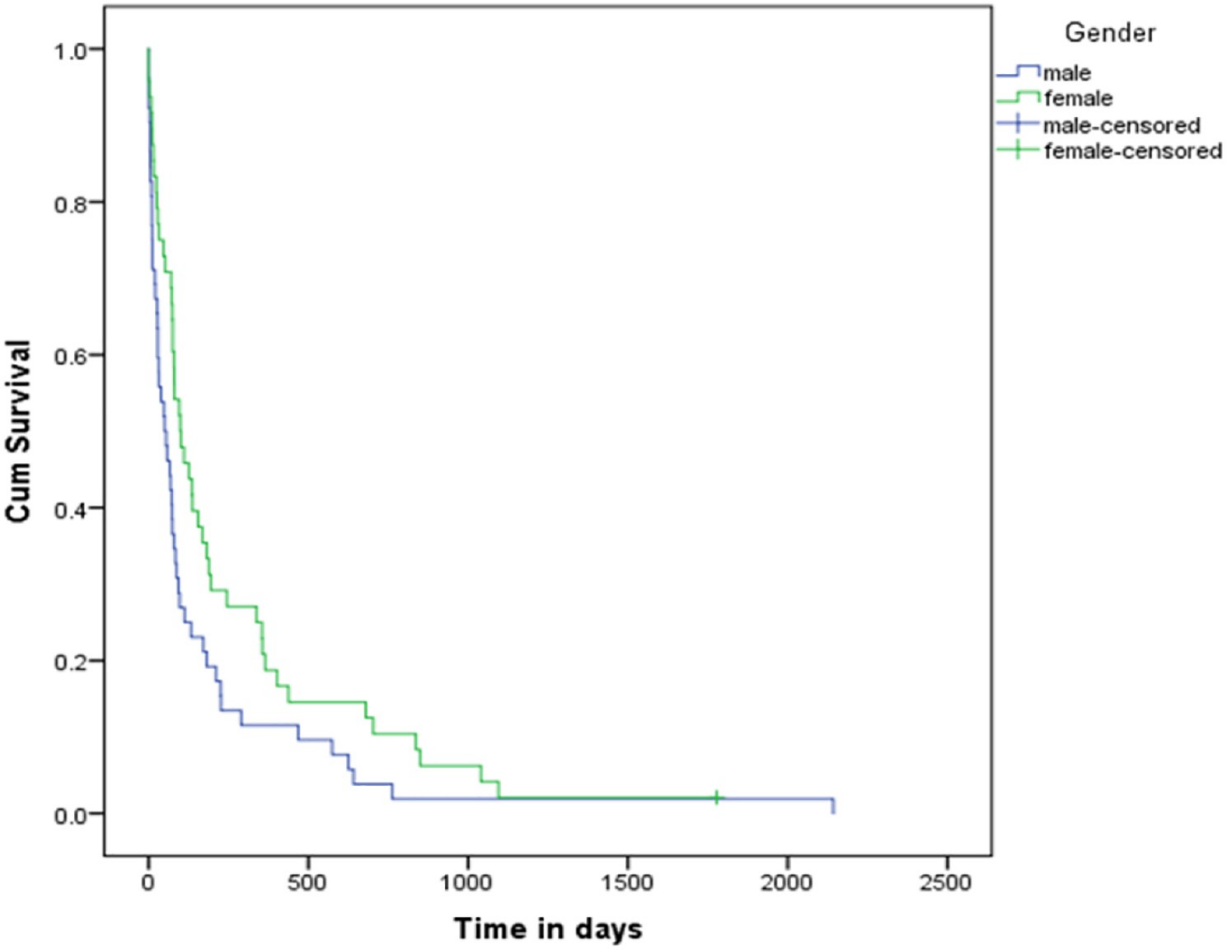
Survival curve for male and female participants with pancreatic cancer.

Median survival of pancreatic cancer patients after diagnosis was 75 days and one-, two- and three-year survival rates were 0.15, 0.07 and 0.02. The median survival of men with pancreatic cancer after diagnosis was 50 days, and their one-, two-, and three-year survival rates were 0.115, 0.038, and 0.038, respectively. Also, the median survival of women with pancreatic cancer after diagnosis was 99 days, and their one-, two-, and three-year survival rates were 0.188, 0.104, and 0.042, respectively. The results of the log-rank test showed that the distribution of survival after pancreatic cancer is different between women and men, and this difference is statistically significant Figs 2 and 3, Table 5.

**Table 5:** Survival rate by gender in PC participants.

Gender	Estimates	Std. Error	95% Confidence Interval	Log Rank
Female	251.146	51.081	151.028 to 351.264	**0.041**
Male	156.135	46.222	65.539 to 246.730	

The median survival of patients under 50 years of age with pancreatic cancer was 79 days after diagnosis and their one- and two-year survival rates were 0.167 and 0.056, respectively. Also, the median survival of patients aged 50 to 59 with pancreatic cancer was 99 days after diagnosis and their one- and two-year survival rates were 0.278 and 0.111, respectively, and the median survival of patients older than and equal to 60 years with pancreatic cancer was 52 days after diagnosis and their one- and two-year survival rates were 0.043 and 0.043, respectively. The log-rank test results showed that the distribution of survival after pancreatic cancer is different among age groups and this difference is statistically significant Table 6.

**Table 6:** Survival rate by age groups in PC participants.

Age groups	Estimates	Std. Error	95% Confidence Interval	Log rank
<50	222.667	72.786	80.006 to 365.327	0.002
50–59	330.111	81.747	169.887 to 490.335	
≥60	93.087	18.618	56.595 to 129.579	

## Discussion

The incidence of PC was 0.2% in Iran, a middle SDI (Socio-demographic Index) country with Age-standardized incidence rate (ASR) of 11.12 per 100,000 person-years. In high SDI countries, the incidence of PC is 3–4 times higher [12]. Due to the aging of the population and their lifestyles, which increase exposure to PC risk factors [4, 13], or because these nations have more access to imaging facilities and a better level of public health awareness [13], PC is more prevalent in high SDI countries.

In our study, incidence density per 100,000 person-year was higher in men. also men had lower survival rates significantly compared to women but the male gender, with a 0.853 HR, was not considered a risk factor for PC. In this line, a study from Israel considered the HR of gender in PC not statistically significant, and PC incidence did not differ between men and women [14]. In contrast, some studies showed that the prevalence of PC in men is higher than in women [15, 16]. Evidence has suggested that this difference can be in terms of the differences in exposure to lifestyle, environmental risk factors, and genetic differences [15] or higher levels of steroid hormones in the women as a protective factor compared to men [16].

Age was shown to be the primary risk factor for PC; people with ≥60 years old age had higher incidence density, lower survival rate, and a 6.564 HR compared to <50 years old. Among other research, PC was shown to be more prevalent among the elderly; 90% of new cases were found in adults over the age of 55, with the frequency being highest in the seventh and eighth decades of life. The most critical risk factor for PC development occurred within these age ranges [4, 16–18].

In our study’s population, 75% of patients with PC were of Turkmen ethnicity, similar to the GCS where 74.29% of participants were Turkman. Still, ethnicity was not observed as a risk factor statistically. In other studies, race was an essential and well-known risk factor for PC. In all united states (US), except Hawaii, the prevalence, and death of PC were higher in African-Americans rather than whites [15, 16, 17].

None of the blood groups was a risk factor for PC. Studies showed that blood groups except O were the risk factors for PC [17, 19]. Results obtained from a large cohort study suggested that the risk of PC is higher in people with A, B, and AB blood groups. Genetic studies showed that the ABO locus on chromosome 9q34 is associated with the risk of PC [15]. The role of blood group in PC may be due to the interaction between the immune system and ABO blood group, which may induce cancer or metastasis [16].

One of our findings in this study included that 27% of the patients were current or former smokers and smoking not identified as a risk factor (HR = 1.656). An OR of 1.2 was found in a research between former smokers and those who had never smoked. Additionally, PC risk rose among smokers who smoked more cigarettes per day and for longer than 50 years [4, 14, 15]. Other studies considered that current smokers had a greater chance of developing PC than non-smoker people [16, 17, 20, 21]. The nicotine in cigarettes is absorbed in the upper digestive tract, and causes genetic mutations in pancreatic cells. The paracrine signaling mechanism, which depends on the microenvironment, is adversely affected by nicotine [9, 16, 17]. There is a relative dose-response relationship between the number of cigarettes and the duration of use [14, 18]. On the other hand, some studies did not observe a relationship between smoking and PC [15, 22]. Shakeri et al. in Iran showed that cigarette smoking did not associate with risk of PC [23].

Opium was not a risk factor for PC in our research (HR = 1.01). In contrast, a prospective research in Iran found that long-term opium usage tripled the incidence of PC compared to never using drugs [24]. Studies observed that opium consumption increased the risk of PC [6, 24, 25]. Furthermore, studies reported a strong relationship between the amount of opium consumption and the risk of developing PC, so this risk is significantly higher in heavy users [24, 25]. Recently, the opium has been recognized as a carcinogen, and mentioned opium pyrolysates have genotoxic properties [6, 24].

We have not observed DM as a risk factor for the incidence of PC, possibly in terms of the low sample size for DM (only 5 cases). Thus, another study from Iran showed DM increased risk of PC (adjusted OR:1.99) [26]. The US National Cancer Institute (NCI) estimated that DM raised the risk of getting PC by 1.8 times, and since chronic DM is now more common worldwide, its impact on the illness is predicted to grow [4]. Many studies considered PC chance increased by DM in the following years [7–9, 14–17, 19, 20, 21, 27, 28]. In addition to being a risk factor for PC, new-onset DM can be an early symptom of this disease [17, 18, 21, 29].

In our study, BMI was not a risk factor for PC development. Therefore, like our study, no relationship between BMI and PC risk was observed in two pooled studies from the Asian community [30]. Still, many studies showed that obesity and high BMI increased the risk of PC [4, 9, 13, 15, 19, 21].

In our study, alcohol consumption not observed as a risk factor for PC. Considering the illegality of alcohol consumption in Iranian society, the data on alcohol consumption may not be accurate. Therefore, it is essential to evaluate the non-significant connection between this risk factor and PC incidence with care. Extensivealcohol use was linked to an elevated risk of PC, according to a meta-analysis research by Wang et al. It is crucial that people who consume muchalcohol are at risk of chronic pancreatitis, which itself can be one of the causes of PC [9]. Regarding alcohol, and its relationship with PC, studies are limited to its heavy consumption [14, 21, 27]. On the other hand, in a case-control study, was not observe the relationship between alcohol consumption and the risk of PC [19].

In our study, none of the people diagnosed with PC during 15 years had an FH of PC, whereas, in the total GCS population, 37 people reported an FH of PC, that none of them developed PC in follow-up years. Several studies showed that people with an FH of PC have a higher risk of PC [7, 19, 20, 27]. Other studies considered that an FH of PC in a first-degree family member did not significantly increase PC risk. Another Iranian research found that having a first-degree family member with a cancer history raised PC risk by 10.9% [26]. Van Tran T et al. found no correlation between an FH of PC and the risk of PC [14], which is similar to what we found.

Our study had some limitations, including a small number of PC cases; therefore, the assessment of risk factors may need a larger population and longer follow-up. We did not measure the amount of alcohol, opium, and cigarette consumption and their relation with PC development. Our study population was not from different areas of Iran; it was limited to the Golestan province.

## Conclusion

The current research shows that 0.2% of GCS participants have a PC. Age was the primary risk factor for PC after adjactment. People at risk for PC were exposed to risk variables more often as they aged.
